# Medical device availability under system degradation: a dependency-based framework for adaptive responses and risk-informed decision-making

**DOI:** 10.3205/dgkh000672

**Published:** 2026-09-01

**Authors:** Manuel Döhla, Peter Dorzweiler, Nico T. Mutters, Katharina Schötz, Franziska Vaupel, Florian Helm

**Affiliations:** 1 Department of Preventive and Veterinary Medicine; Bundeswehr Healthcare Command, Koblenz, Germany; 2 University of Bonn, University Hospital Bonn, Institute for Hygiene and Public Health, Bonn, Germany; 3 Department of Microbiology and Hospital Hygiene, Bundeswehr Central Hospital Koblenz, Koblenz, Germany

**Keywords:** healthcare resilience, disaster medicine, healthcare logistics, infection prevention and control, medical devices, adaptive reprocessing, austere environments

## Abstract

The availability of medical devices (MDs) is commonly viewed as a challenge related to procurement, logistics, or reprocessing. However, recent conflicts, disasters, pandemics, and attacks on healthcare systems have shown that the availability of MDs depends on a broader network of interdependent resources. Disruption to these dependencies may not only compromise the acquisition of new MDs, but also the continued use and reprocessing of existing ones.

This recommendation conceptualises MD availability as a system-dependent healthcare capability. Five key dependency domains are proposed to describe the resources and functions required to maintain device availability in degraded conditions. Based on these dependencies, a seven-component adaptive response framework (7R) is presented: Relocate, Rebuy, Rebuild, Rethink, Reduce, Reuse, and Reprocess.

Adaptive reprocessing is examined as a representative high-dependency response strategy because it necessitates the coordinated interaction of multiple dependency domains while simultaneously introducing risks relating to infection prevention, functionality, operations, and governance. Rather than eliminating risk, adaptive measures redistribute risk across domains.

The proposed framework links dependencies, adaptive response options, reprocessing functions and residual risk, supporting preparedness planning and operational decision-making when conventional standards can no longer be fully maintained.

## Abstract (de)

Die Verfügbarkeit von Medizinprodukten (MP) wird gewöhnlich als Herausforderung von Einkauf, Logistik oder Aufbereitung verstanden. Allerdings zeigen moderne Konflikte, Katastrophen, Pandemien und Angriffe auf Gesundheitssysteme, dass die Verfügbarkeit von MP von einem großen Netzwerk voneinander wechselwirkenden Ressourcen abhängt. Fehlen diese Ressourcen, kann sich das nicht nur negativ auf die Anschaffung neuer MP auswirken, sondern auch die weitere Nutzung und Aufbereitung vorhandener MP beeinflussen.

Diese Arbeit konzipiert die Verfügbarkeit von MP als eine systemabhängige Fähigkeit eines Gesundheitssystems. Es werden fünf wichtige Bereiche vorgeschlagen, um die Ressourcen und Funktionen zu beschreiben, die notwendig sind, um die Verfügbarkeit von MP unter degradierten Bedingungen sicherzustellen. Auf Basis dieser Abhängigkeiten wird ein Framework vorgestellt, das sieben mögliche adaptive Antworten beschreibt (7R): räumliche Verlagerung, Nachkaufen, Herstellen, die Nutzung optimieren, die Nutzung einschränken, erneute Nutzung ohne Aufbereitung und behelfsmäßige Aufbereitung.

Behelfsmäßige Aufbereitung wird als anspruchsvollste Antwortoption gesondert untersucht, weil sie komplexe Abhängigkeiten aufweist, die aufeinander abgestimmt werden müssen. Gleichzeitig entstehen hierdurch neue Risiken hinsichtlich Infektionsschutz, Funktionalität, Einsatzführung und Kontrolle. Anstatt diese Risiken vollständig zu eliminieren, können adaptive Antworten lediglich eine Verschiebung von Restrisiken zwischen diesen Risikoarten erreichen.

Das vorgestellte Framework verbindet Abhängigkeiten, adaptive Antworten, Einzelschritte der Aufbereitung von MP und entstehende Risiken. Es unterstützt dadurch die Notfallplanung und die operative Entscheidungsfindung, wenn gewohnte Standards nicht länger vollständig aufrechterhalten werden können.

## Introduction

Medical devices (MDs) are essential components of modern healthcare systems and depend on complex supply, maintenance, and reprocessing structures to remain available and functional. Armed conflicts, disasters, attacks on healthcare infrastructure, pandemics, and large-scale supply chain disruptions can compromise these structures and threaten the availability of critical MDs. Recent conflicts and humanitarian crises have repeatedly demonstrated the vulnerability of healthcare infrastructure, while the COVID-19 pandemic exposed major weaknesses in global medical supply and logistics networks [1], [2], [3], [4], [5].

Disruptions affecting MD availability may arise from damaged infrastructure, shortages of water, energy, personnel, consumables, transportation capacity, or interruptions in manufacturing and procurement networks. Such failures may force healthcare systems to adapt their use of existing MDs, modify clinical workflows, or implement alternative strategies to maintain operational capability. For the purpose of this recommendation, system degradation is defined as the partial loss, disruption, or insufficiency of infrastructure, material, human, organisational, or assurance-related resources required to maintain healthcare functions and medical device availability. Although MD reprocessing represents one potential response to resource scarcity, it constitutes only one element within a broader spectrum of adaptive measures [6], [7], [8], [9].

This recommendation addresses this gap by conceptualising medical device availability as a system-dependent healthcare capability [10]. We propose

1. five dependency domains,
2. a seven-component adaptive response framework (7R), and
3. a structured, function-based approach to adaptive reprocessing under degraded conditions (Figure 1 (Fig. 1)).

The framework is intended to support preparedness planning, operational decision-making, and future research across military, disaster, humanitarian, and resource-constrained healthcare settings.

## Method

This recommendation was developed based on a targeted narrative review of the literature on MD reprocessing, healthcare preparedness, disaster medicine, austere healthcare environments, and healthcare service continuity under resource-constrained conditions. Relevant publications addressing reprocessing requirements, infrastructure dependencies, adaptive response strategies, and risk management were identified through literature searches and expert review.

The identified concepts were synthesized into a dependency-based framework describing factors influencing MD availability under degraded conditions. The framework was further informed by the authors' professional experience in hospital hygiene, infection prevention and control, military medicine, healthcare preparedness, and medical support operations in resource-constrained environments, including operational and advisory roles within the German Armed Forces medical service.

The resulting recommendation represents a structured expert synthesis intended to support decision-making regarding MD availability when standard reprocessing systems are disrupted or unavailable.

## Results

### Dependency domains of medical device availability

MD availability depends on a network of interdependent resources, support functions, and governance mechanisms [10]. Although the relative importance of individual dependencies differs between reusable and single-use devices, all MDs ultimately rely on a combination of identical requirements. For operational planning, these dependencies can be grouped into five domains comprising fifteen core dependencies (Table 1 (Tab. 1)).

#### Infrastructure

Infrastructure forms the physical foundation of MD availability and reprocessing capability. Three core dependencies can be distinguished: water, energy, and space. Water is essential for cleaning, disinfection, and sterilisation, while electrical energy supports reprocessing equipment and associated systems. Suitable space is required to separate contaminated and clean workflows, store supplies, and conduct reprocessing activities safely [11], [12], [13]. System degradation may disrupt these dependencies through facility damage, utility outages, overcrowding, or environmental constraints. Such disruptions can compromise both the availability of MDs and the ability to maintain reusable MDs through reprocessing. Recent conflicts, such as the war in Ukraine and the conflict in Gaza, have repeatedly demonstrated the vulnerability of healthcare infrastructure, including the destruction of power and water systems as well as medical supply chains and reprocessing facilities [14], [15], [16], [17]. As a result, infrastructure often represents the first and most fundamental limiting factor in resource-constrained healthcare environments. 

#### Material

Material resources comprise the equipment, chemicals, and packaging materials required to maintain MD availability. Reusable MDs depend on reprocessing equipment, cleaning and disinfection agents, sterilisation systems, and packaging materials. Single-use MDs, while less dependent on reprocessing resources, remain vulnerable to shortages of consumables, replacement products, and supporting materials [3], [4]. System degradation may interrupt manufacturing, procurement, transportation, or storage, resulting in shortages that directly affect clinical capability. Material dependencies are closely linked to supply chains, energy availability, and personnel competency. Consequently, shortages of critical materials may not only impair reprocessing activities but also reduce the availability, functionality, or safety of MDs across the healthcare system [1].

#### Human

Human resources encompass both the availability of personnel and the competencies required to safely manage MDs. MD availability depends on sufficient staffing, technical expertise, training, and procedural knowledge. Reprocessing activities require personnel capable of performing cleaning, disinfection, inspection, sterilisation, documentation, and quality assurance tasks according to established standards [18], [19]. System degradation may reduce workforce availability through injury, illness, displacement, fatigue, or competing operational demands. Simultaneously, disruptions may increase the need for improvisation and adaptive decision-making, placing greater demands on individual competency. Human dependencies include the ability to maintain safe practices under resource-constrained conditions. Inadequate staffing or training may compromise both medical device availability and patient safety [4].

#### Organisation

Organisational dependencies comprise the procedural and logistical structures required to maintain medical device MD availability [10]. Key elements include supply chains, transportation networks, and waste management systems. Procurement and logistics ensure the delivery and distribution of MDs, spare parts, consumables, chemicals, and packaging materials. Waste management supports the safe handling and disposal of contaminated materials, thereby preventing environmental contamination and maintaining operational continuity [6]. System degradation may disrupt these systems through damaged transport routes, supply shortages, communication failures, or reduced disposal capacity. Such disruptions can compromise both the acquisition of new devices and the continued use of reusable MDs. Consequently, MD availability depends not only on local resources but also on the functionality of broader support systems that connect healthcare operations with external sources of supply and disposal [1], [3].

### Assurance

Assurance functions encompass the mechanisms required to verify, document, and govern the safe availability and use of MDs. Key elements include monitoring, documentation, and governance. Monitoring activities provide evidence that processes are functioning as intended, while documentation supports traceability, accountability, and continuity of operations. Governance includes policies, standards, regulatory requirements, and local decision-making structures that define acceptable levels of risk and guide adaptive measures [20], [21]. Unlike material or infrastructural dependencies, assurance functions do not directly create medical device availability but provide confidence in the processes used to maintain it. System degradation may compromise monitoring systems, record keeping, oversight mechanisms, and regulatory compliance, increasing uncertainty and operational risk. Consequently, assurance functions represent a critical but often overlooked dependency domain that supports both medical device availability and patient safety [22].

#### Dependency failure

Failure of a single dependency may impair individual functions, whereas disruption of multiple domains may threaten overall MD availability. The relative contribution of individual domains varies according to MD type and supply strategy. While single-use devices primarily depend on procurement and logistics systems, reusable devices additionally require reprocessing capabilities supported by all five dependency domains. Understanding these dependencies provides the basis for identifying adaptive response options under system-degraded conditions [3], [23].

### 7R Framework

#### From dependency failure to adaptive response

System degradation may compromise one or multiple dependency domains simultaneously, resulting in reduced MD availability. The consequences of such failures vary according to the affected dependencies, device characteristics, clinical requirements, and operational context.

Adaptive responses represent practical strategies for sustaining MD availability when conventional systems can no longer be fully maintained. The 7R framework is an inductive extension of a previously described 3R framework [24]. The 7R framework is not intended as a strict sequential escalation pathway. Strategies on the left side (relocate, rebuy, rebuild) generally preserve higher standards and are preferred when feasible. Strategies on the right side (reuse, reprocess) become increasingly relevant when external resources are severely limited, albeit at the cost of higher residual risk. The seven strategies may be implemented individually or in combination depending on available resources and acceptable levels of risk (Figure 2 (Fig. 2)).

The framework consists of the following seven complementary strategies:

- **Relocate:** Transfer of MD availability functions to an alternative location capable of maintaining required standards,
- **Rebuy** (external replenishment): Replacement of unavailable or exhausted devices through external procurement,
- **Rebuild:** Local manufacture, reconstruction, or fabrication of MDs or components.
- **Rethink:** Qualitative optimisation of MD utilisation and clinical workflows,
- **Reduce:** Quantitative reduction of MD consumption,
- **Reuse:** Continued use of an MD without formal reprocessing,
- **Reprocess:** Adaptive restoration of device usability through cleaning, disinfection, sterilisation and related measures under degraded conditions.

#### From adaptive response to adaptive reprocessing

The 7R framework describes complementary strategies for maintaining MD availability under system degradation. Depending on the nature and severity of the disruption, multiple strategies may be implemented simultaneously. Whenever feasible, relocation, procurement, local manufacture, optimisation of utilisation, or demand reduction may preserve device availability while avoiding additional technical and regulatory challenges. Reuse may further extend the availability of existing resources but may introduce important safety and compliance concerns [25].

Among the seven adaptive response options, reprocessing occupies a unique position. Unlike other strategies, adaptive reprocessing requires the coordinated interaction of multiple dependency domains, including infrastructure, materials, personnel, organisational systems, and assurance functions [26]. As a result, reprocessing represents one of the most resource-intensive and risk-sensitive approaches for maintaining MD availability. At the same time, it may provide a critical capability when alternative response options are unavailable or insufficient. Understanding how reprocessing can be adapted under degraded conditions therefore remains essential for operational preparedness and resilience. The following chapter examines adaptive reprocessing along the medical device reprocessing chain, with particular emphasis on required process functions, adaptive measures, and associated risks [7], [27], [28].

### Adaptive reprocessing

#### Risk-based reprocessing requirements

Not all MDs require the same level of reprocessing. According to the Spaulding classification, reprocessing requirements are determined by the intended use of a device and the associated risk of infection transmission. Non-critical devices contact intact skin and generally require cleaning, whereas semi-critical devices contact mucous membranes or non-intact skin and typically require cleaning and disinfection. Critical devices enter sterile body sites or the vascular system and therefore require the highest level of reprocessing, including sterilisation [26], [29]. Under system-degraded conditions, these differences become particularly important because not all process steps may remain fully available. Adaptive reprocessing strategies should therefore be guided by the minimum reprocessing depth required for safe device use (Table 2 (Tab. 2)). Consequently, the objective of adaptive reprocessing is not necessarily to preserve every element of the conventional process chain, but to maintain the core functions required to achieve an appropriate level of device safety according to the intended use and risk classification of the device [26], [30].

#### Adaptive measures along the reprocessing chain

The MD reprocessing chain consists of multiple process steps, each fulfilling a distinct function that contributes to device safety and availability (Table 3 (Tab. 3)) [20], [31]. Under system-degraded conditions, conventional procedures may no longer be fully achievable because of limitations affecting critical dependencies. In such circumstances, the objective of adaptive reprocessing is not necessarily to replicate standard reprocessing procedures but to preserve the core function of each process step to the greatest extent possible. The following sections outline potential adaptive measures and associated residual risks for individual process steps. The described measures are intended as conceptual examples rather than technical recommendations and should always be interpreted within the context of device classification, intended use, available resources, and acceptable risk [28], [30].

#### Collection and transport

Collection and transport ensure the separation, containment, and safe handling of contaminated MDs prior to reprocessing (core function). System degradation may limit access to designated collection systems, transport containers, or dedicated transport routes. Potential adaptive measures include simplified collection procedures, temporary containment systems, designated transport schedules, or the use of locally available transport solutions. Contaminated devices should remain clearly identifiable and physically separated from clean devices throughout handling and transport (adaptive measures). Inadequate segregation or transport may increase contamination risks for personnel, patients, equipment, and the environment and may compromise subsequent reprocessing activities (residual risks).

#### Pre-cleaning

Pre-cleaning prevents drying and fixation of organic contamination between device use and definitive reprocessing. Maintaining moisture and removing gross contamination facilitates subsequent cleaning and reduces the risk of persistent soil (core function) [32]. System degradation may limit access to dedicated pre-cleaning products, water, or trained personnel. Potential adaptive measures include immediate removal of visible contamination, use of locally available moistening methods, prioritisation of heavily contaminated devices, and minimisation of delays before further processing (adaptive measures). Delayed or inadequate pre-cleaning may increase cleaning difficulty, reduce subsequent process effectiveness, and elevate contamination risks throughout the reprocessing chain (residual risks).

#### Cleaning

Cleaning removes organic and inorganic contamination from medical device surfaces and constitutes the foundation of all subsequent reprocessing activities [29]. Effective cleaning is essential because residual contamination may impair both disinfection and sterilisation processes (core function). System degradation may restrict access to water, detergents, automated cleaning systems, or trained personnel. Potential adaptive measures include manual cleaning methods, prioritisation of critical devices, simplified cleaning workflows, or use of alternative cleaning resources where appropriate. Regardless of the method employed, visible contamination should be removed to the greatest extent possible before further processing (adaptive measures). Incomplete cleaning may compromise the effectiveness of downstream reprocessing steps, increase infection risks, and reduce confidence in overall device safety. Because cleaning underpins the entire reprocessing chain, failures at this stage may propagate throughout subsequent processes (residual risks).

#### Disinfection

Disinfection reduces microbial burden to a level appropriate for the intended use of the device and serves as a critical step for many semi-critical devices (core function) [29]. System degradation may limit access to validated disinfectants, automated systems, or controlled processing conditions. Potential adaptive measures include prioritisation of available disinfectants, use of alternative validated products, adjustment of workflow priorities, or implementation of simplified disinfection procedures consistent with available resources (adaptive measures). Reduced disinfection effectiveness may increase the likelihood of pathogen transmission and compromise patient and personnel safety (residual risks).

#### Inspection and functional testing

Inspection and functional testing identify damage, wear, residual contamination, and functional deficiencies that may compromise device performance or safety. In addition to infection prevention considerations, inspection provides assurance that a device remains fit for its intended purpose (core function). System degradation may limit access to testing equipment, reference materials, illumination, magnification, or experienced personnel. Potential adaptive measures include simplified visual inspection procedures, prioritisation of critical device functions, use of alternative testing approaches, and focused assessment of high-risk components. Where comprehensive testing is not feasible, available resources should be directed toward functions most relevant to patient safety (adaptive measures). Undetected damage, contamination, or malfunction may compromise device performance, increase procedural risks, and adversely affect patient outcomes despite otherwise successful reprocessing (residual risks).

#### Packaging

Packaging provides a barrier against recontamination and enables the maintenance of device integrity and sterility during storage and transport. The required level of packaging depends on device classification, intended use, and the subsequent reprocessing pathway (core function). System degradation may result in shortages of sterile barrier systems, wrapping materials, indicator systems, or sealing equipment. Potential adaptive measures include prioritisation of available packaging resources for critical devices, simplification of packaging configurations, utilisation of locally available barrier materials, or immediate use of reprocessed devices to reduce storage requirements. Depending on the operational context, packaging requirements may therefore need to be adapted to available resources and intended device use (adaptive measures). Reduced packaging performance may increase the risk of recontamination, compromise storage stability, and limit traceability. In addition, deviations from established packaging standards may create governance and quality assurance challenges (residual risks).

#### Sterilisation

Sterilisation aims to achieve sterility for critical medical devices intended for contact with sterile tissues, body cavities, or the vascular system (core function) [20]. System degradation may restrict access to validated sterilisation equipment, utilities, consumables, or monitoring systems. Potential adaptive measures include use of alternative sterilisation technologies, prioritisation of critical devices, simplification of sterilisation workflows, or deployment of mobile and improvised sterilisation capabilities where feasible. The chosen approach should seek to preserve the intended function of sterilisation while acknowledging operational constraints (adaptive measures). Uncertainty regarding sterilisation performance may increase infection prevention risks [26], reduce confidence in device safety, and create substantial governance and accountability challenges (residual risks).

#### Storage and redistribution

Storage and redistribution preserve device integrity, sterility where applicable, and operational readiness until subsequent use (core function). System degradation may limit storage capacity, environmental controls, inventory systems, or transport capabilities. Potential adaptive measures include prioritization of critical devices, reduction of storage times, decentralised distribution approaches, and adaptation of inventory practices to available resources (adaptive measures). Inadequate storage conditions may compromise device status, increase contamination risks, and reduce operational availability (residual risks).

#### Documentation and traceability

Documentation and traceability provide accountability, process verification, and the ability to reconstruct device histories throughout the reprocessing cycle (core function). System degradation may impair electronic systems, record keeping, labelling processes, or oversight mechanisms. Potential adaptive measures include simplified documentation procedures, paper-based systems, abbreviated records, or prioritisation of traceability for high-risk devices and processes (adaptive measures). Reduced documentation may limit traceability, complicate incident investigation, impair quality assurance, and increase governance and regulatory risks associated with adaptive reprocessing (residual risks).

### From adaptive measures to residual risk: risk domain, trade-offs and decision-making

Adaptive measures may help preserve the core functions of individual reprocessing steps despite system degradation. However, adaptation rarely eliminates risk entirely. Instead, residual risks remain and may affect infection prevention and control (IPC), device functionality, operational performance, or governance processes to varying degrees. Understanding these trade-offs is therefore essential for informed decision-making under degraded conditions [27], [30], [33]. The following chapter proposes a conceptual framework for assessing and balancing these residual risks across adaptive reprocessing strategies.

#### Risk domains

Adaptive reprocessing measures may preserve medical device availability under system degradation but may also introduce residual risks. These risks can be grouped into four domains: IPC, functional, operational, and governance risks. IPC risks relate to contamination, pathogen transmission, and patient safety. Functional risks concern device integrity, performance, and fitness for intended use. Operational risks affect healthcare capability, resource utilisation, and continuity of services. Governance risks encompass documentation, traceability, accountability, regulatory compliance, and oversight [32], [34].

The relative importance of these domains varies across reprocessing steps and operational contexts. While some adaptive measures predominantly affect a single domain, others may influence several domains simultaneously. Consequently, evaluation of adaptive reprocessing strategies requires consideration not only of their potential benefits but also of the distribution of residual risks across these four domains [27].

#### Risk redistribution and trade-offs

As illustrated in Table 4 (Tab. 4), the dominant residual risk domains differ considerably across the reprocessing chain. While adaptive measures affecting cleaning, disinfection, and sterilisation primarily influence infection prevention and control risks, measures targeting inspection and functional testing predominantly affect device performance and patient safety. Packaging and documentation, in contrast, are closely associated with governance-related risks, including traceability, accountability, and regulatory compliance.

Importantly, adaptive measures rarely eliminate risk entirely. Instead, they redistribute risk across domains. Measures intended to preserve medical device availability may reduce operational risks while simultaneously increasing infection prevention, functional, or governance risks. Consequently, adaptive reprocessing should not be viewed as a binary choice between safe and unsafe practice, but rather as a process of balancing competing risks under constrained conditions [25], [27].

Published examples of adaptive reprocessing approaches illustrate this principle. Reprocessing strategies for filtering face piece respirators, for example, may improve operational availability during shortages while introducing uncertainties regarding filtration performance, process validation, or regulatory compliance [24], [35]. Similarly, improvised reprocessing approaches developed for resource-constrained or field environments may preserve critical capabilities but often require acceptance of increased residual risks in other domains. The objective of adaptive reprocessing is therefore the transparent identification and management of residual risk within the operational context [26].

Table 4 (Tab. 4) represents a conceptual impact matrix based on the authors’ expert synthesis. The indicated impact levels are not empirically quantified but serve to illustrate typical risk redistribution patterns and to support structured risk communication in operational settings.

#### Decision-making under system degradation

No adaptive response strategy is entirely risk free. Under system-degraded conditions, decision-makers are frequently required to balance competing priorities, including IPC, device functionality, operational capability, and regulatory requirements. The optimal strategy therefore depends not only on technical feasibility but also on the clinical context, available resources, and acceptable levels of residual risk.

This framework is intended to support structured decision-making rather than prescribe specific solutions. Dependency domains help identify limiting factors, the 7R framework provides a spectrum of adaptive response options, and the conceptual impact matrix highlights potential risk distributions associated with adaptive reprocessing measures. Together, these elements may assist healthcare organisations in selecting context-appropriate strategies for maintaining medical device availability when conventional standards can no longer be fully achieved.

## Discussion

MD availability is often approached as a problem of procurement, logistics, or reprocessing. However, recent conflicts, disasters, attacks on healthcare facilities, and large-scale supply chain disruptions have demonstrated that MD availability depends on a broader network of interdependent resources and support functions [1], [2], [3], [4], [5], [14], [15], [16], [17]. Disruptions affecting infrastructure, material, humans, organisation, or assurance may compromise both the acquisition of new devices and the continued use of existing ones. The proposed framework therefore conceptualises MD availability as a system-dependent healthcare capability rather than solely a logistical challenge.

The proposed dependency domains highlight that adaptive reprocessing represents only one possible response to system degradation. Reprocessing has traditionally received considerable attention because it may extend the usability of existing devices during shortages. However, reprocessing itself depends on multiple infrastructure, material, human, organisational, and assurance-related resources. Consequently, maintaining MD availability may often be achieved more effectively through alternative adaptive strategies such as relocation, external procurement, local production, optimisation of utilisation, or demand reduction. The 7R framework is intended to support structured consideration of these options rather than promote reprocessing as a preferred solution.

Adaptive measures rarely eliminate risk entirely. Instead, they redistribute residual risks across domains, requiring decision-makers to balance competing priorities [20], [24], [25], [26], [27], [35]. Measures intended to preserve operational capability may increase infection prevention, functional, or governance-related risks, whereas strict adherence to conventional standards may compromise operational capability if resources become unavailable. Decision-making under system-degraded conditions therefore requires transparent consideration of competing risks and priorities rather than a binary distinction between acceptable and unacceptable practice. The proposed risk domains provide a conceptual structure for communicating and balancing these trade-offs.

The practical relevance of individual dependency domains and adaptive response options is likely to vary according to the nature of the disruption. For example, armed conflicts may primarily affect infrastructure, transportation networks, and supply chains, whereas pandemics may predominantly challenge workforce availability and material resources. Natural disasters may simultaneously impair multiple dependency domains. Consequently, the framework should not be interpreted as a prescriptive model but as a flexible decision-support tool that may assist preparedness planning and operational decision-making across a range of military, disaster, humanitarian, and resource-constrained healthcare environments.

## Limitations

This recommendation has several limitations. The proposed framework represents a structured expert synthesis informed by the literature and the authors’ professional experience rather than an empirically validated model. The relative importance of individual dependencies, adaptive response strategies, and risk domains may vary substantially between healthcare systems, device types, and operational contexts. Furthermore, the impact matrix is intended to illustrate conceptual patterns of risk redistribution and does not provide quantitative risk estimates. Future empirical studies should evaluate the applicability of the framework in real-world healthcare disruptions and explore methods for operationalising dependency assessment and risk-informed decision-making under system-degraded conditions.

## Conclusion

System degradation threatens MD availability through the disruption of multiple interdependent resources, processes, and governance functions. Maintaining healthcare capability under such conditions therefore requires adaptive approaches that extend beyond conventional procurement and reprocessing strategies.

This recommendation proposes a framework consisting of five dependency domains, seven adaptive response options (7R), and a structured approach to adaptive reprocessing based on core process functions. By linking dependencies, adaptive response options, reprocessing functions, and risk domains, the proposed framework provides a practical conceptual foundation to enhance the resilience of MD availability in an era of increasing infrastructure threats. It supports preparedness planning, operational decision-making, and offers a basis for future empirical validation in military, disaster, humanitarian, and other resource-constrained healthcare environments.

Future empirical studies and field validations are needed to test and refine this framework in real-world military, disaster, and humanitarian settings.

## Notes

### Authors’ ORCIDs

- Döhla M: https://orcid.org/0000-0001-8029-5264
- Dorzweiler P: https://orcid.org/0009-0000-8812-0354
- Mutters NT: https://orcid.org/0000-0002-0156-9595
- Schötz K: https://orcid.org/0009-0000-4826-4951
- Vaupel F: http://orcid.org/0009-0002-2555-3077

### Acknowledgments

The authors thank Dr. Daniela Cortnum, Christina Döhla, and Dirk Hiller for their valuable expert input and discussions regarding field medicine, logistics and MP reprocessing standards.

### Funding

None. 

### AI usage statement

Generative artificial intelligence tools (OpenAI GPT-5.5-based ChatGPT and DALL·E image generation models; OpenAI, San Francisco, CA, USA) were used to support graphical concept development and language refinement. All scientific content and final editorial decisions were made by the authors.

### Competing interests

The authors declare that they have no competing interests.

## Figures and Tables

**Table 1:**
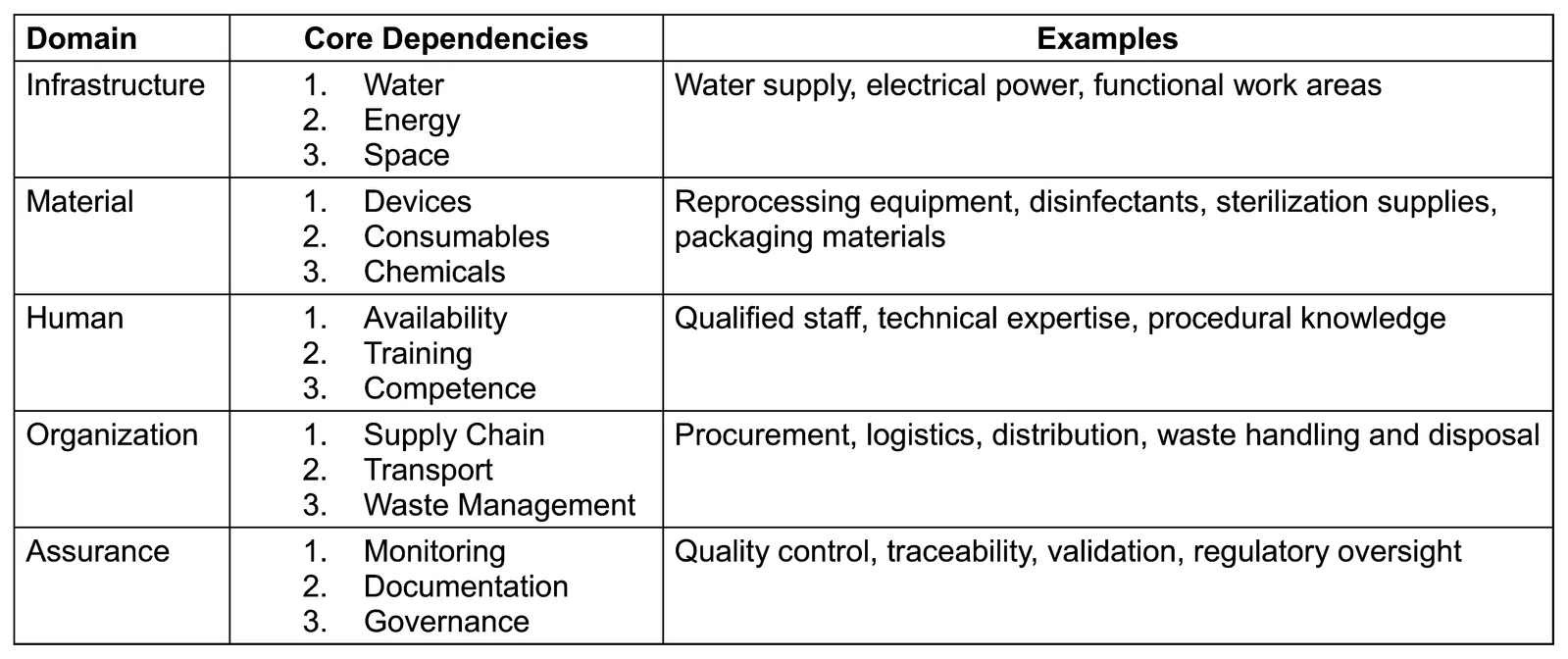
Dependency domains of medical device availability under system degradation

**Table 2:**
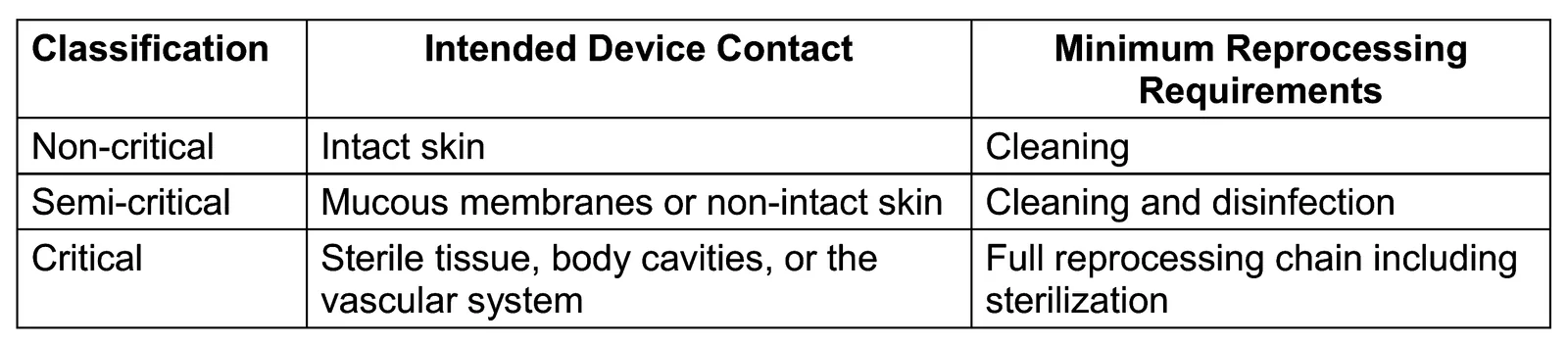
Risk-based reprocessing requirements according to the Spaulding classification. The Spaulding classification defines the minimum reprocessing depth required to achieve an appro-priate level of device safety. Under system-degraded conditions, adaptive reprocessing measures should therefore be guided by device classification and intended use. While critical devices gen-erally require the complete reprocessing chain, selected non-critical and semi-critical devices may remain suitable for use following a reduced number of process steps.

**Table 3:**
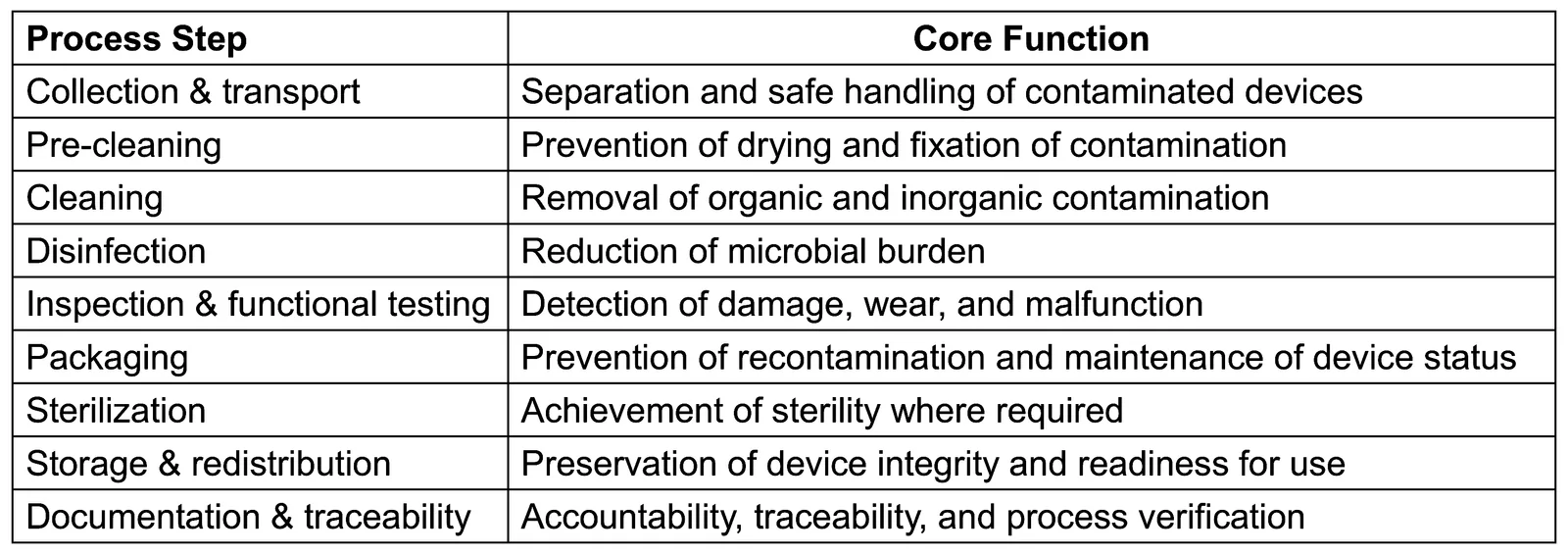
Core functions of the medical device reprocessing chain under system-degraded condi-tions

**Table 4:**
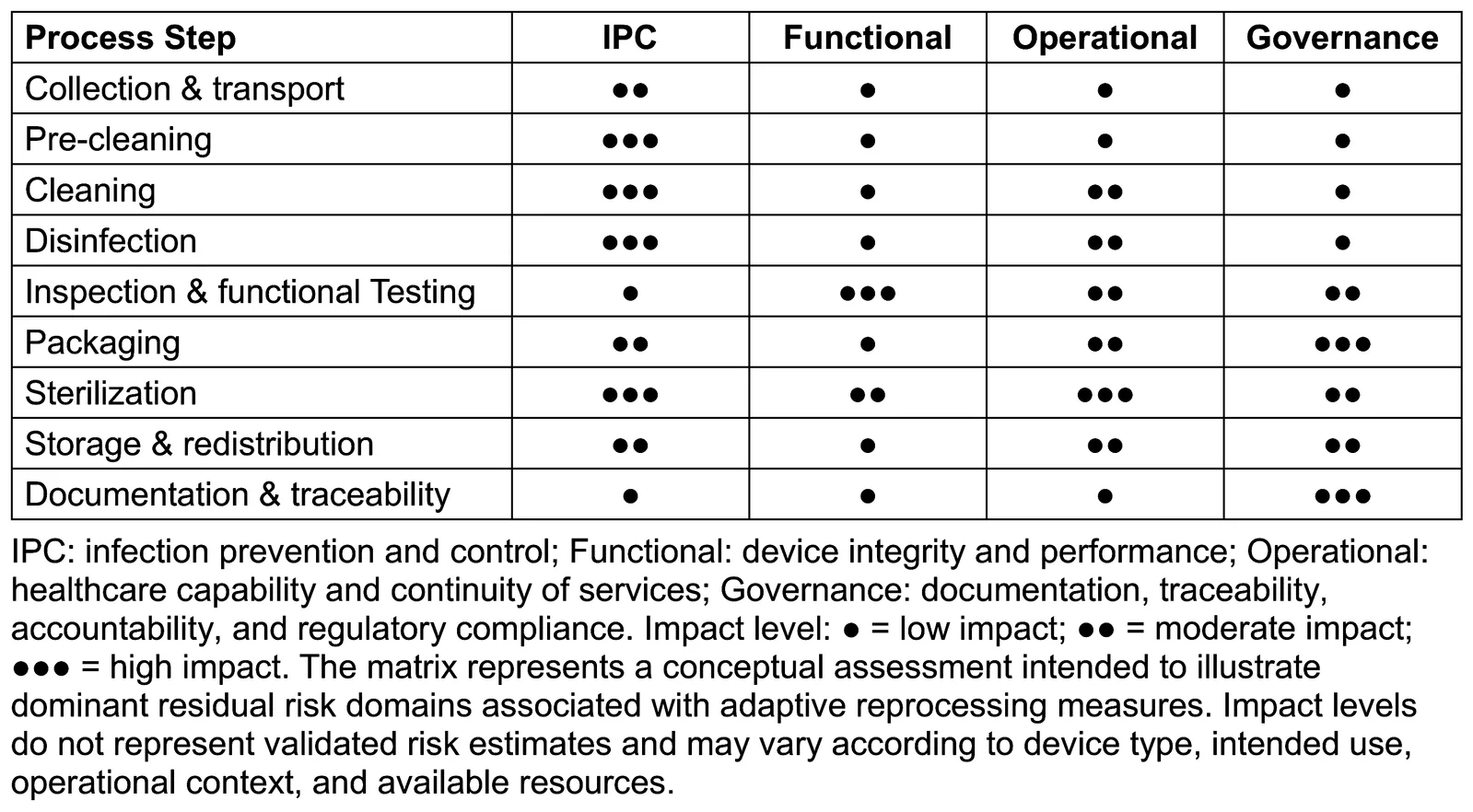
Conceptual impact matrix for adaptive reprocessing under system degradation.

**Figure 1:**
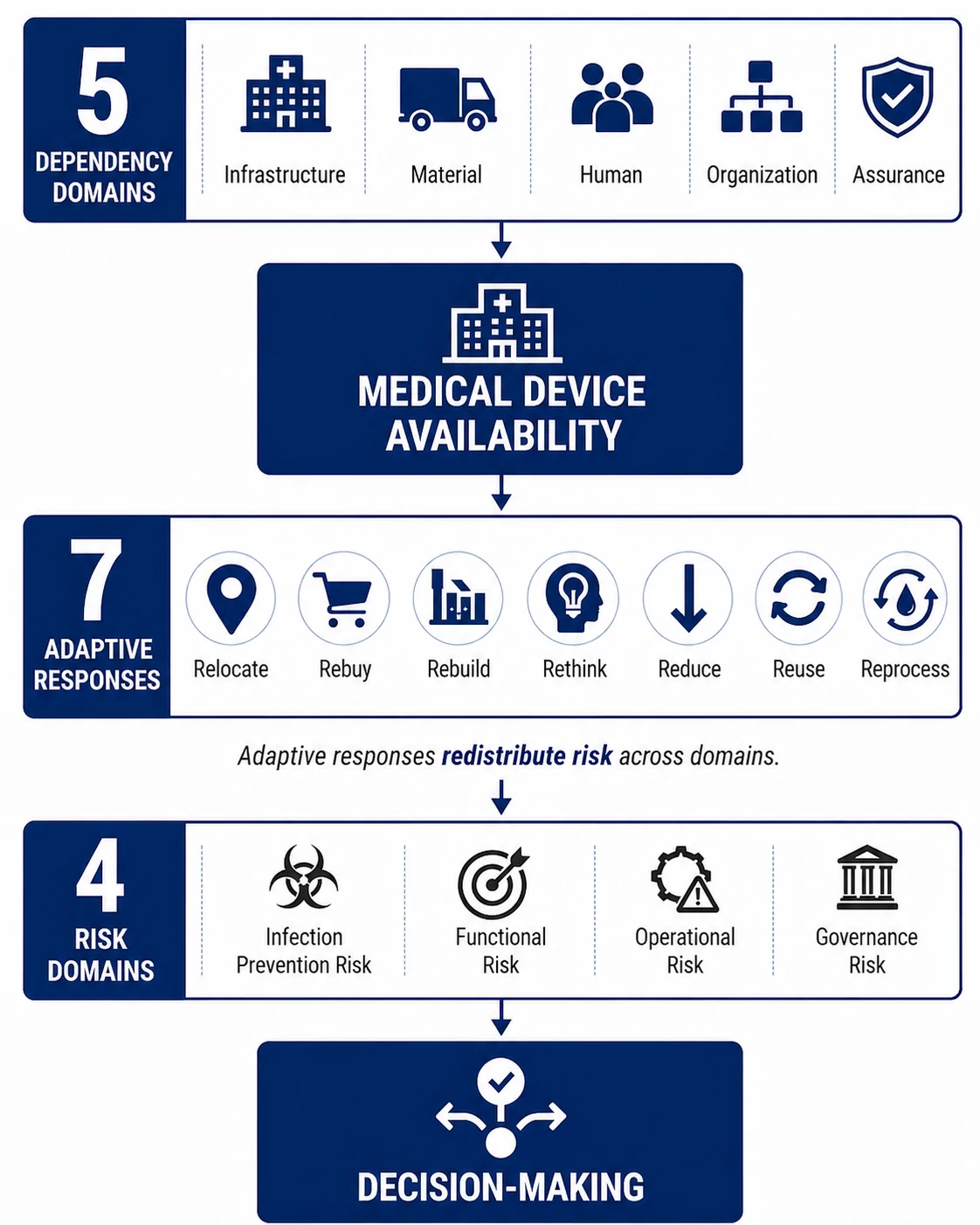
The proposed MP Availability framework with five dependency domains, a seven-component adaptive response framework (7R), and a structured, function-based approach to adaptive reprocessing under degraded conditions.

**Figure 2:**
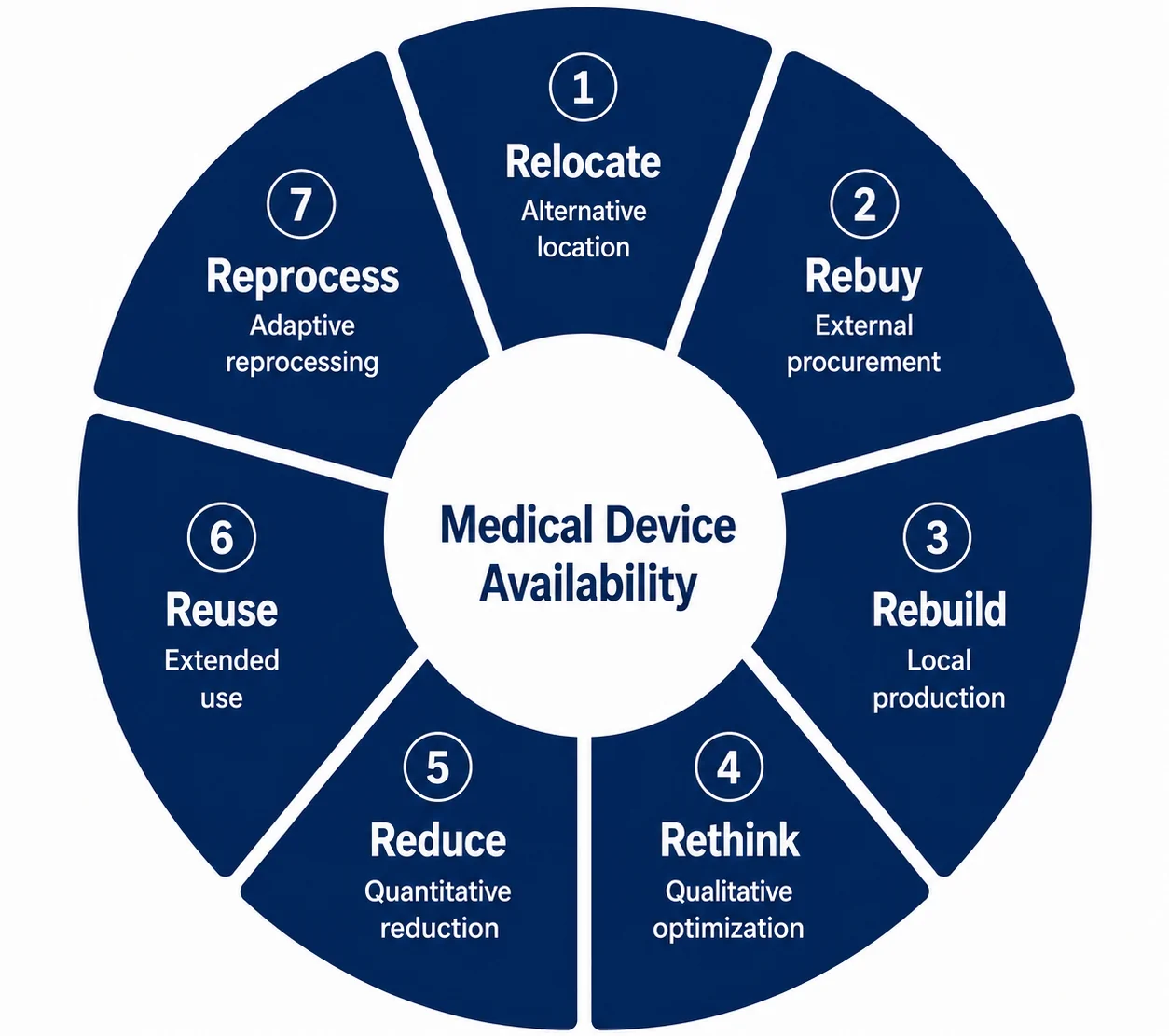
The 7R Adaptive Response Framework. The framework conceptualizes seven adaptive response options for maintaining medical device availability under system degradation. The strategies (Relocate, Rebuy, Rebuild, Rethink, Reduce, Reuse, and Reprocess) represent complementary rather than sequential approaches and may be implemented simultaneously according to operational requirements, resource availability, and acceptable risk.

## References

[1] Bhaskar S, Tan J, Bogers MLAM, Minssen T, Badaruddin H, Israeli-Korn S, Chesbrough H (2020). At the Epicenter of COVID-19-the Tragic Failure of the Global Supply Chain for Medical Supplies. Front Public Health.

[2] Bown CP (2022). How COVID-19 medical supply shortages led to extraordinary trade and industrial policy. Asian Economic Policy Review.

[3] Miller FA, Young SB, Dobrow M, Shojania KG (2021). Vulnerability of the medical product supply chain: the wake-up call of COVID-19. BMJ Qual Saf.

[4] Thai L, Beleche T, Adetunji O (2025). Understanding the Impact and Costs Associated with Medical Device Shortages During the COVID-19 Pandemic on Providers, Health Systems, Patients, and Manufacturers.

[5] Safeguarding Health in Conflict Coalition (2026). Care in the crosshairs: Violence Against Health Care in Conflict 2025.

[6] Shore C, Brown L, Hopp WJ (2022). Causes and Consequences of Medical Product Supply Chain Failures.

[7] Josephs-Spaulding J, Singh OV (2021). Medical Device Sterilization and Reprocessing in the Era of Multidrug-Resistant (MDR) Bacteria: Issues and Regulatory Concepts. Front Med Technol.

[8] FDA (2025). Medical Device Supply Chain and Shortages.

[9] Phillips SJ, Knebel RA, Johnson KJ (2009). Mass Medical Care with Scarce Resources: The Essentials. AHRQ Publication No. 09-0016.

[10] World Health Organization (2024). Building health system resilience to public health challenges; guidance for implementation in countries.

[11] Kamm L, Hagen RM, Mutters NT, Schmithausen RM, Weppler R, Döhla M (2025). Drinking water and sanitation safety planning for medical facilities: An iInnovative PoU approach for a water system description using Ecomaps. Environments.

[12] Krause S, Joel E, Schaum C, Bäumer J, Rücker N, Wienand I, Sturm C, Jahn-Mühl B, Geiger M, Fekete A, van der Heijden S, Heinzel C, Sandholz S (2024). Water safety planning for healthcare facilities for extreme events. J Water Health.

[13] Centers for Disease Control and Prevention (2022). Healthcare Water System Repair. Natural Disasters and Severe Weather.

[14] World Health Organization (2026). Attacks on Ukraine’s health care increased by 20% in 2025.

[15] International Committee of the Red Cross (2015). Health Care In Danger.

[16] Lawry LL, Janvrin M, Korona-Bailey J, Betancourt C, Maddox J, Apilado KP, Juman L, Mani V, Kanagaratnam A, Amowitz Z, Hamm TE, Berezyuk O, Koehlmoos TP (2025). Health System Organization and Logistics of Trauma Care Since the Russian Invasion of Ukraine: A Qualitative Assessment. Inquiry.

[17] Ali M, Rehman IU, Lee KS, Ming LC (2025). Gaza's health emergency: impact of armed conflict and its global health repercussions. Global Health.

[18] Surgical Directions (2025). Urgent Sterile Processing Workforce Challenges Impacting Surgical Safety. Surgical Directions..

[19] Leach R (2026). Curing the Sterile Processing Department Staffing Crisis.

[20] World Health Organization Decontamination and reprocessing of medical devices for health care facilities: aide-memoire.

[21] Amaral C, Paiva M, Rodrigues AR, Veiga F, Bell V (2024). Global regulatory challenges for medical devices: Impact on innovation and market access. Appl Sci.

[22] Heinrichmann F, Herrmann O, Stroebe M, Schwabedissen M (2024). Quality considerations in disaster recovery: A case study.

[23] Tarver M (2025). Medical Device Supply Chain Vulnerabilities and the Public Health Impact They Have on Our Most Vulnerable Patients.

[24] Döhla M, Becker E, Granzer H, Gubajdulin E, Langer P, Mutschnik C, Scheid PL, Wiemann J, Hagen RM (2024). Successful establishment and evaluation of a reprocessing concept via steam at 105°C for FFP masks in hospitals in case of logistic shortages. J Occup Environ Hyg.

[25] Wurmb TE, Schade J, Schräder SJ, Meybohm P, Weibel S, Kippnich M (2024). Adjustment of medical standards in disaster, crises and war: a scoping review of the literature. BMJ Public Health.

[26] Bailey-Monje U, Becker E, Funke S, Gubajdulin E, Hagen RM, Kirmse G, Lütz K, Mutters NT, Schmithausen RM, Weppler R, Döhla M (2025). Provisional Reprocessing of Medical Devices in Field Hospitals: Evaluation of Chemical Approaches for Feasibility and Effectiveness. Disaster Med Public Health Prep.

[27] Antonini MJ, Plana D, Srinivasan S, Atta L, Achanta A, Yang H, Cramer AK, Freake J, Sinha MS, Yu SH, LeBoeuf NR, Linville-Engler B, Sorger PK (2021). A Crisis-Responsive Framework for Medical Device Development Applied to the COVID-19 Pandemic. Front Digit Health.

[28] Shelton S, Hamm J, Olatosi B, Johnson RO (2018). Recovery of Surgical Equipment Sterile Processing During a Floodwater Boil Advisory. Disaster Med Public Health Prep.

[29] Centers for Disease Control and Prevention, Infection Control (2024). A Rational Approach to Disinfection and Sterilization.

[30] Rowan NJ, Kremer T, McDonnell G (2023). A review of Spaulding's classification system for effective cleaning, disinfection and sterilization of reusable medical devices: Viewed through a modern-day lens that will inform and enable future sustainability. Sci Total Environ.

[31] European Commission (2026). Reprocessing of devices.

[32] (2021). ISO 17664-1. Processing of health care products — Information to be provided by the medical device manufacturer for the processing of medical devices. Part 1: Critical and semi-critical medical devices.

[33] (2019). ISO 14971. Medical devices — Application of risk management to medical devices.

[34] Mahmoud T, Balachandran W, Altayyar S (2024). Advancing sustainable healthcare technology management: Developing a comprehensive risk assessment framework with a fuzzy analytical hierarchy process, integrating external and internal factors in the Gulf Region. Sustainability.

[35] Vaupel F, Fengler I, Mutters NT, Scheid P, Sib E, Weppler R, Döhla M (2024). Investigation of Three Different UV-C Irradiation Schemes for Bacterial Decontamination of FFP2 Masks to Make Them Reusable. Disaster Med Public Health Prep.

